# A phosphoinositide hub connects CLE peptide signaling and polar auxin efflux regulation

**DOI:** 10.1038/s41467-023-36200-0

**Published:** 2023-01-26

**Authors:** Qian Wang, A. Cecilia Aliaga Fandino, Moritz Graeff, Thomas A. DeFalco, Cyril Zipfel, Christian S. Hardtke

**Affiliations:** 1 Department of Plant Molecular Biology, University of Lausanne, CH-1015 Lausanne, Switzerland; 2 Institute of Plant and Microbial Biology, University of Zurich, Zurich-Basel Plant Science Center, CH-8008 Zurich, Switzerland; 3 Present Address: Department of Biology, Western University, London, Canada

**Keywords:** Plant development, Plant cell biology, Phosphoinositol signalling

## Abstract

Auxin efflux through plasma-membrane-integral PIN-FORMED (PIN) carriers is essential for plant tissue organization and tightly regulated. For instance, a molecular rheostat critically controls PIN-mediated auxin transport in developing protophloem sieve elements of *Arabidopsis* roots. Plasma-membrane-association of the rheostat proteins, BREVIS RADIX (BRX) and PROTEIN KINASE ASSOCIATED WITH BRX (PAX), is reinforced by interaction with PHOSPHATIDYLINOSITOL-4-PHOSPHATE-5-KINASE (PIP5K). Genetic evidence suggests that BRX dampens autocrine signaling of CLAVATA3/EMBRYO SURROUNDING REGION-RELATED 45 (CLE45) peptide via its receptor BARELY ANY MERISTEM 3 (BAM3). How excess CLE45-BAM3 signaling interferes with protophloem development and whether it does so directly or indirectly remains unclear. Here we show that rheostat polarity is independent of PIN polarity, but interdependent with PIP5K. Catalytically inactive PIP5K confers rheostat polarity without reinforcing its localization, revealing a possible PIP5K scaffolding function. Moreover, PIP5K and PAX cooperatively control local PIN abundance. We further find that CLE45-BAM3 signaling branches via RLCK-VII/PBS1-LIKE (PBL) cytoplasmic kinases to destabilize rheostat localization. Our data thus reveal antagonism between CLE45-BAM3-PBL signaling and PIP5K that converges on auxin efflux regulation through dynamic control of PAX polarity. Because second-site *bam3* mutation suppresses root as well as shoot phenotypes of *pip5k* mutants, CLE peptide signaling likely modulates phosphoinositide-dependent processes in various developmental contexts.

## Abstract (web summary)

In this paper the authors demonstrate that auxin efflux in the phloem vascular tissue of plants is determined by antagonism between CLE peptide signaling pathways and phosphoinositide kinases.

## Introduction

Meristems are the growth apices of plants and are metabolic sinks whose growth is sustained by phloem sap delivery through the early protophloem tissue^1^. The stem cell niche in the primary root meristem of *Arabidopsis thaliana* (*Arabidopsis*) continuously produces two distinct protophloem poles that flank a central xylem axis^2^. Within each pole, the protophloem sieve element (PPSE) cell file is central because developing PPSEs organize the formation of immediately adjacent tissues^3^, and because the differentiated PPSEs constitute the terminal conduit for phloem sap into the meristem^1^. Thus, PPSE development is essential for root meristem maintenance and growth^1,4,5^. This is evident in *brx* or *pax* mutants, which display PPSE differentiation failures and reduced root growth vigor^5,6^. The plasma-membrane-associated BRX and PAX proteins interact and constitute a molecular rheostat that regulates PIN-mediated cellular auxin efflux^6,7^. This protophloem-specific machinery is thought to promote auxin canalization into developing PPSEs to coordinate their development and promote their timely differentiation^7,8^.

Rheostat regulation of auxin efflux in developing PPSEs emerges from dynamic interactions. Briefly, BRX inhibits PAX-mediated PIN activation at low cellular auxin, but is displaced from the plasma membrane as auxin levels rise and PAX is activated via the 3-phosphoinositide-dependent protein kinases  (PDKs)^6,8,9^. Consequently, PIN-mediated auxin efflux increases, and as cellular auxin levels drop, BRX eventually re-associates with the plasma membrane. This interplay is reinforced by transcriptional feedbacks and association of the rheostat proteins with PIP5K^10^. In *Arabidopsis*, PIP5K1 and PIP5K2 are the dominant phosphoinositide 5-kinases and are expressed throughout the root^11,12^. Loss-of-function *pip5k1 pip5k2 (pip5k)* double mutants display severely impaired growth and various developmental deficiencies^11,12^, including root protophloem differentiation defects that are the hallmark of the rheostat mutants, *brx* and *pax*^10^. PIP5K is generally found at the plasma membrane and in the nucleus^11,12^, yet it is strongly polar in developing PPSEs, where it is recruited to the center of the rootward plasma membrane by the PPSE-specific BRX and PAX proteins^10^. Because PIP5K catalyzes the formation of phosphatidylinositol-4,5-bisphosphate (PI(4,5)P2), a key determinant for polar PAX plasma-membrane-association^10,13,14^, this reinforces the localization of all three proteins^10^. Moreover, their combined action results in distinct, PPSE-specific subcellular patterning of rootward polarized PINs such as the dominant PIN1, which may reflect PI(4,5)P2-stimulated PIN endocytosis and is thought to be necessary for properly integrated sieve element differentiation^10,11,15,16^. In summary, the current model suggests that the rheostat dynamically modulates PIN activity as well as abundance, resulting in low PIN levels in the center and high PIN levels in the periphery of the rootward plasma membrane^8,10,17^. This PPSE-specific ‘donut’-like PIN pattern is quantitatively disrupted in *brx*, *pax* and *pip5k* mutants, which instead more frequently display the ‘pancake’-like even PIN distribution throughout the plasma membrane as is typically observed in other cell files^10^.

The *brx* mutant phenotype is suppressed by second-site loss-of-function mutations in BAM3, the CLE45 receptor^18,19^. Consistently, the protophloem-specific CLE45-BAM3 pathway is hyperactive in *brx* mutants^18,20^ and external application of synthetic CLE45 peptide suppresses protophloem differentiation^5,19,21^. It remains unclear however how CLE45-BAM3 signaling interferes with PPSE development, and whether it does so directly or indirectly. Here we investigated whether CLE45-BAM3 signaling intersects with the activity or polarity of the rheostat module, and thereby auxin efflux regulation.

## Results

### Rheostat polarity is independent of PIN polarity

**Fig. 1:**
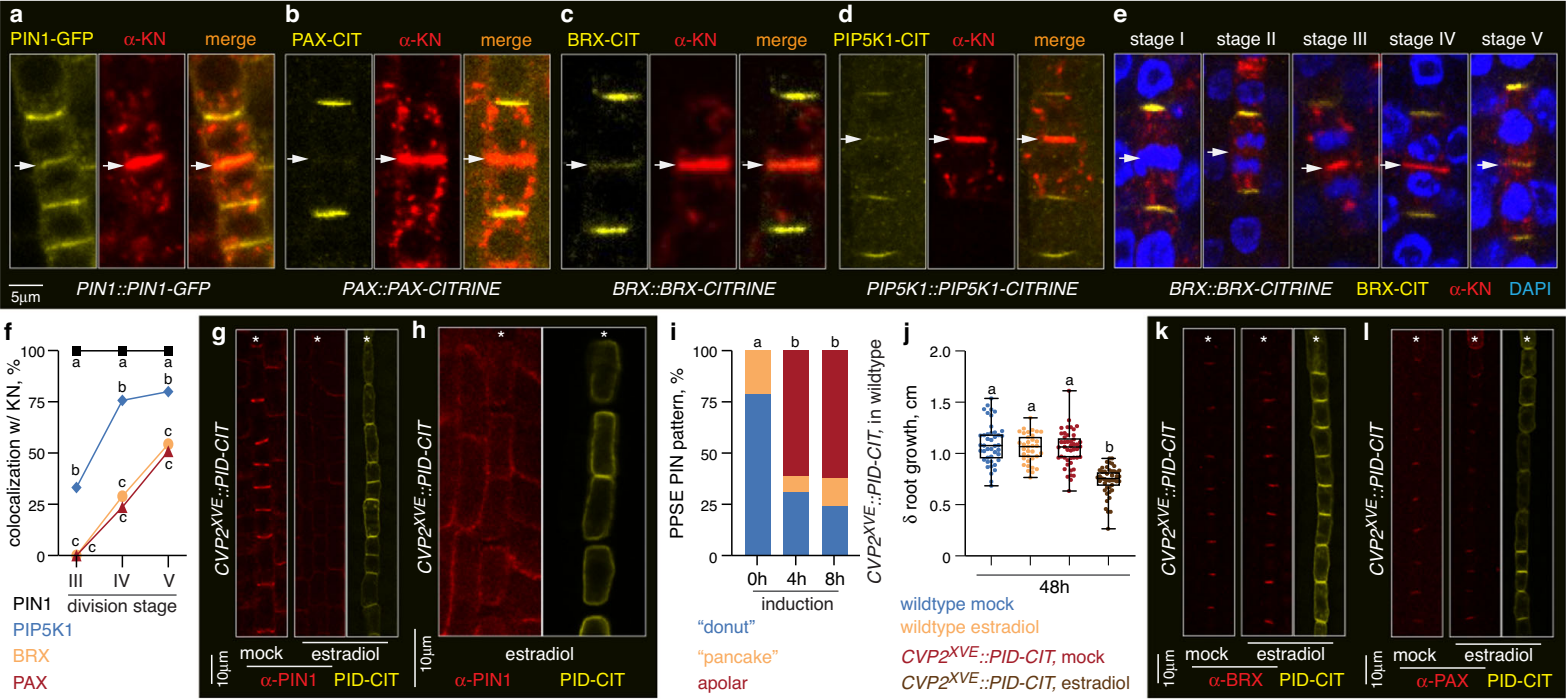
Polar auxin efflux in developing protophloem sieve elements (PPSEs) is required for root development. **a**–**d** Confocal microscopy images of PIN1, PAX, BRX and PIP5K1 -GFP or -CITRINE (CIT) fusion proteins (yellow fluorescence) expressed under their native promoters (*PIN1::PIN1-GFP*; *PAX::PAX-CIT*; *BRX::BRX-CIT*; *PIP5K1::PIP5K1-CIT*), detected by anti-GFP immunostaining in dividing PPSEs. The (nascent) cell plates were detected by simultaneous anti-KN immunostaining (red fluorescence). **e** Confocal images of BRX-CIT protein detected by anti-GFP immunostaining (yellow fluorescence), (nascent) cell plates detected by anti-KN immunostaining (red fluorescence), and DNA by DAPI staining (blue fluorescence), in dividing PPSEs at different stages of the cell division cycle. **f** Quantification of PIN1, PAX, BRX and PIP5K1 colocalization with the nascent cell plate at different stages as illustrated in (**e**), representing 37–277 dividing cells surveyed in 44–332 PPSE cell files. Percentages indicate the fraction of cells of a given stage in which a given protein was detected at the cell plate. **g** Confocal images of PIN1 localization (anti-PIN1 immunostaining, red fluorescence) in developing PPSE cell files (asterisks) of 5-day-old transgenic seedlings expressing a PINOID (PID) kinase CITRINE fusion protein under control of an estradiol-inducible *COTYLEDON VASCULAR PATTERN 2* promoter (*CVP2*^*XVE*^*::PID-CIT*), in mock conditions or after 8 h PID-CIT (anti-GFP immunostaining, yellow fluorescence) induction on 5 micromolar estradiol. **h** As in (**g**), after root “squashing” to separate individual cells. **i** Scoring of PIN polarity and subcellular patterning in developing PPSEs of 5-day-old *CVP2*^*XVE*^*::PID-CIT* transgenics, following PID-CIT induction on 5 micromolar estradiol. Class “apolar”: even PIN1 localization around the cell; class “donut”: polar PIN localization that displays the PPSE-specific abundance minimum in the center; class “pancake”: polar PIN localization that displays even distribution throughout the rootward plasma membrane. *n* = 20–31 roots, 198–297 PPSEs per time point; Statistically significant differences (lower case letters) were determined by Fisher’s exact test, *p* < 0.001. **j** Root growth of wildtype and *CVP2*^*XVE*^*::PID-CIT* transgenics, measured 48 h after transfer of 5-day-old seedlings onto mock or 5 micromolar estradiol media. *n* = 35–49 roots; Statistically significant differences were determined by ordinary one-way ANOVA, *p* < 0.0001. **k**, **l** As in (**g**), with anti-BRX (**k**) or anti-PAX (**l**) immunostaining. Arrows in (**a**–**e**) indicate the position of the cell plate, asterisks in (**g**, **h**, **k**, **l**) mark the PPSE cell files. Box plots display 2nd and 3rd quartiles and the median, bars indicate maximum and minimum. See Source Data for raw values and sample numbers. Source data are provided as a Source Data file.

In dividing PPSEs, PIN1 is already present in early cell plates (Fig. 1a), unlike BRX or PAX (Fig. 1b, c), which only appear upon completion of cytokinesis^10^. In high throughput immunostaining quantifications of colocalization with the cell plate marker KNOLLE (KN)^22^, we found that PIP5K appears earlier than BRX or PAX (Fig. 1d), but still after PIN1 (Fig. 1e, f). To determine whether the polarity of the rheostat module thus depends on PIN1, we employed the PINOID (PID) kinase, which can alter PIN polarity^23,24^. PPSE-specific induction of PID readily triggered PIN1 depolarization (Fig. 1g–i), which was accompanied by impaired root growth and protophloem development (Fig. 1j and Supplementary Fig. 1a–d). However, PID induction altered neither BRX nor PAX polarity or abundance (Fig. 1k, l, and Supplementary Fig. 1e, f). In summary, these experiments reiterate the central role of the protophloem in root meristem maintenance^2,5,25^ and directly demonstrate the importance of controlled polar auxin efflux for PPSE development^8^. They also show that polarity of the rheostat proteins is independent of PIN polarity.

### Rheostat and PIP5K polarity display an internal hierarchy and interdependence

Polar plasma-membrane-association of the rheostat proteins and PIP5K is largely interdependent, as the steady state abundance of each is reduced in the other mutant backgrounds^6,10^. To explore the dynamics of this interplay, we introduced PPSE-specific inducible transgenes for *BRX*, *PAX* and *PIP5K1* into various mutants (Supplementary Fig. 2a–i). This allowed us to monitor the interdependence of the protein localizations uncoupled from the rather low endogenous expression levels, because the induced transgenic fusion proteins were by comparison over-expressed. These experiments revealed an internal hierarchy, wherein PIP5K is required for efficient BRX as well as PAX localization (Supplementary Fig. 2c, f), with the caveat that transgene induction in the *pip5k* background was possibly sub-optimal due to the strong protophloem phenotype. Conversely, PIP5K1 localization strongly depends on BRX but not on PAX (Supplementary Fig. 2g, h), and BRX and PAX depend on each other to a lesser degree (Supplementary Fig. 2b, e). Mapping of the interaction between the three proteins by yeast-two-hybrid assays further indicated that the kinase domain of PAX interacts with the C-terminal ‘BRX-domains’ of BRX (Supplementary Fig. 3a, b). The BRX C-terminus also interacts with PIP5K1 and PIP5K2 (Supplementary Fig. 3c). Furthermore, PIP5K1 and 2 interact with the PAX N-terminus (Supplementary Fig. 3d). These observations suggest that the BRX, PAX and PIP5K proteins tightly assemble through mutual interactions.

Compared to other AGC1 clade kinases, such as the prototypical D6 PROTEIN KINASE (D6PK), the PAX N-terminus is unique^13,26^, and unlike PAX, D6PK could not interact with PIP5K (Supplementary Fig. 3e). However, D6PK could interact with the BRX-domains (Supplementary Fig. 3b), consistent with partial rescue of subcellular PIN patterning defects in *pax* mutants upon D6PK expression under control of the *PAX* promoter^10^. To also test the role of PAX kinase activity, we created a PAX point mutant variant (M440G; ‘ciPAX’) that can be conditionally inactivated by the ATP analog, 4-amino-1-tert-butyl-3-(1′-naphthyl)pyrazolo[3,4-d]pyrimidine (NAPP)^27,28^. The ciPAX variant fully complemented the *pax* mutant in normal conditions, but not in the presence of NAPP (Supplementary Fig. 4a, b). Moreover, auxin-induced BRX plasma-membrane-dissociation was impaired upon NAPP treatment (Supplementary Fig. 4c), consistent with the key role of a PAX target phosphosite in BRX auxin-response^15^. These experiments demonstrate that the specific action of PAX in protophloem development involves its unique N-terminus and its capacity to thereby efficiently recruit PIP5K with the help of BRX.

### PIP5K can function as a scaffold

**Fig. 2:**
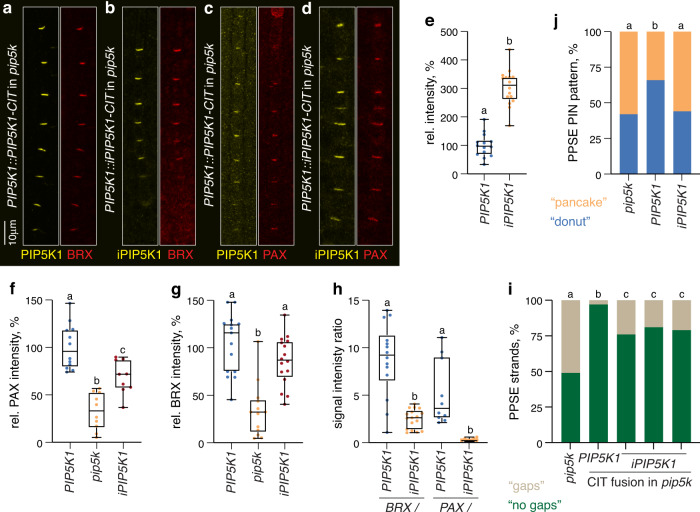
PIP5K can serve as a scaffold in developing PPSEs. **a**–**d** Confocal microscopy images of BRX (a-b; anti-BRX immunostaining, red fluorescence) and PAX (**c**, **d**; anti-PAX immunostaining, red fluorescence) in developing PPSEs of 5-day-old *pip5k* seedlings expressing either PIP5K1 (**a**, **c**) or iPIP5K1 (**b**, **d**) CIT fusion proteins (anti-GFP immunostaining, yellow fluorescence) under control of the *PIP5K1* promoter (*PIP5K1::PIP5K1-CIT* and *PIP5K1::iPIP5K1-CIT*, respectively). **e** Relative abundance of the PIP5K1-CIT fusion protein variants (**a**–**d**) as determined by anti-GFP immunostaining. *n* = 14–16 roots, 116–207 PPSEs per genotype; Statistically significant difference (lower case letters) was determined by Student’s *t* test, *p* < 0.0001. **f**, **g** Relative abundance of PAX (**f**) and BRX (**g**) as determined by anti-PAX and anti-BRX immunostaining in developing PPSEs of 5-day-old *pip5k* mutant and *PIP5K1::PIP5K1-CIT* and *PIP5K1::iPIP5K1-CIT* transgenics. (**f**): *n* = 9–12 roots, 6–9 PPSEs per root, 69–82 cells total per genotype; (**g**): *n* = 13–16 roots, 102–178 PPSEs per genotype; Statistically significant differences were determined by ordinary one-way ANOVA, *p* ≤ 0.0042 (**f**) and *p* < 0.0001 (**g**). **h** Ratio of the abundance of BRX and PAX in developing PPSEs of 5-day-old *pip5k* mutants and *PIP5K1::PIP5K1-CIT* and *PIP5K1::iPIP5K1-CIT* transgenics, as determined by anti-BRX/PAX/GFP immunostaining. *n* = 8–17 roots, 102–178 PPSEs per genotype; Statistically significant differences were determined by ordinary one-way ANOVA, *p* ≤ 0.0017. **i** Scoring of PPSE differentiation failures (“gaps”) in 7-day-old *pip5k* mutants and *PIP5K1::PIP5K1-CIT* and *PIP5K1::iPIP5K1-CIT* transgenics. *n* = 47–110 PPSE strands; Statistically significant differences were determined by Fisher’s exact test, *p* ≤ 0.0144. **j** Scoring of subcellular PIN patterning in developing PPSEs of 5-day-old *pip5k* mutants and *PIP5K1::PIP5K1-CIT* and *PIP5K1::iPIP5K1-CIT* transgenics, as determined by anti-PIN1 immunostaining. *n* = 17–29 roots, 97–226 PPSEs per genotype; Statistically significant differences were determined by Fisher’s exact test, *p* < 0.001. Box plots display 2nd and 3rd quartiles and the median, bars indicate maximum and minimum. See Source Data for raw values and sample numbers. Source data are provided as a Source Data file.

Reduced PAX abundance in *brx* mutants is substantially restored by second-site loss-of-function in *COTYLEDON VASCULAR PATTERN 2 (CVP2)*^10^, which encodes a PPSE-specific 5-phosphatase that transforms PI(4,5)P2 into phosphatidylinositol-4-phosphate (PI4P)^29,30^. This is accompanied by restored subcellular PIN patterning, PPSE differentiation and root growth^10,30^, suggesting that upon reduced PI(4,5)P2 conversion to PI4P, PAX is capable of directing PPSE development without BRX. To test whether PAX requires PIP5K, we created a kinase-dead PIP5K1 variant (K536A; ‘iPIP5K1’)^31^. As expected, unlike wildtype PIP5K1^10^, iPIP5K1 could not complement the severe growth defects of *pip5k* mutants (see below). Surprisingly however, iPIP5K1 was still polar localized in PPSEs (Fig. 2a–d) and even appeared more abundant than wildtype PIP5K1 (Fig. 2e). Moreover, iPIP5K1 could still interact with BRX and PAX (Supplementary Fig. 3c, d) and restored both BRX and PAX localization (Fig. 2b and d), although their abundance was lower than in control lines (Fig. 2f, g). Compared to wildtype PIP5K1, BRX and PAX abundance was strongly reduced relative to iPIP5K1 (Fig. 2h), suggesting that iPIP5K1 had lost the capacity to reinforce rheostat localization. Although recruitment of the rheostat proteins by iPIP5K1 partially rescued PPSE differentiation defects (Fig. 2i), it could not significantly restore subcellular PIN1 patterning (Fig. 2j). Thus, both PAX and PIP5K kinase activity are necessary for efficient subcellular PIN patterning in PPSEs, which could mean that PAX-mediated PIN activation also serves as a cue for PIN endocytosis. Indeed, PIN1 endocytosis appeared impaired in *pax* and upon ciPAX inhibition (Supplementary Fig. 4d). Collectively, our data emphasize the importance of the quantitative interplay between the rheostat components and PIP5K, and the synergy between feedback-controlled PIN activation and subcellular patterning. Moreover, our experiments suggest a possible scaffolding function for PIP5K that does not depend on its catalytic activity and might be mediated by interaction with its substrate, PI4P^32^.

### CLE45 peptide signaling interferes with rheostat polarity

**Fig. 3:**
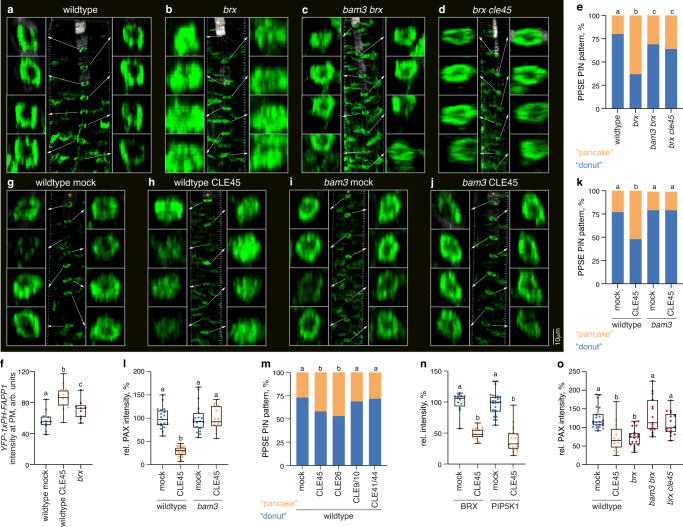
CLE45-BAM3 signaling impairs auxin efflux control in developing PPSEs. **a**–**d** Confocal microscopy images of PIN1 localization (anti-PIN1 immunostaining, green fluorescence) in developing PPSE cell files (calcofluor staining, gray fluorescence; asterisks) of 5-day-old seedlings of indicated genotypes, 3D reconstruction. Center panels: overview; Side panels: magnified images of individual PPSE rootward membranes, with positions indicated by arrows. **e** Scoring of subcellular PIN patterning in developing PPSEs of 5-day-old seedlings exemplified in (**a**–**d**). *n* = 17–23 roots, 141–249 PPSEs per genotype; Statistically significant differences (lower case letters) were determined by Fisher’s exact test, *p* < 0.001. **f** Relative intensity of the constitutively expressed PI4P marker YFP-1xPH-FAPP, determined by live imaging developing PPSEs of 5-day-old wildtype or *brx* seedlings treated with mock or 10 nM CLE45 for 2 h. *n* = 9–15 roots, 72–110 PPSEs per treatment/genotype; Statistically significant differences were determined by ordinary one-way ANOVA, *p* ≤ 0.0334. **g**–**j** Similar to (**a**–**d**), for wildtype or *bam3* seedlings 6 h after transfer onto mock or 10 nM CLE45. **k** Scoring of subcellular PIN patterning in developing PPSEs exemplified in (**g**–**j**). *n* = 16–23 roots, 163–204 PPSEs per genotype; Statistically significant differences were determined by Fisher’s exact test, *p* < 0.001. **l** Relative abundance of PAX, determined by anti-PAX immunostaining in developing PPSEs of 5-day-old wildtype or *bam3* mutant seedlings, treated with mock or 10 nM CLE45 for 6 h. *n* = 15–23 roots, 172–229 PPSEs per genotype/treatment; Statistically significant differences were determined by ordinary one-way ANOVA, *p* < 0.0001. **m** Scoring of subcellular PIN patterning in developing PPSEs of 5-day-old wildtype seedlings, 6 h after transfer onto mock or 10 nM of the indicated CLE peptide, determined by anti-PIN1 immunostaining. *n* = 24–28 roots, 274–314 PPSEs per treatment; Statistically significant differences were determined by Fisher’s exact test, *p* ≤ 0.0018. **n** Relative abundance of BRX and PIP5K1-CIT, determined by anti-BRX or anti-GFP immunostaining in developing PPSEs of 5-day-old seedlings treated with mock or 10 nM CLE45 for 6 h. *n* = 10–28 roots, 115–322 PPSEs per genotype/treatment; Statistically significant differences were determined by ordinary one-way ANOVA, *p* < 0.0001. **o** Relative abundance of PAX, determined by anti-PAX immunostaining in developing PPSEs of 5-day-old seedlings of indicated genotypes, wildtype treated with mock or 15 nM CLE45 for 21 h. *n* = 14–22 roots, 121–184 PPSEs per genotype/treatment; Statistically significant differences were determined by ordinary one-way ANOVA, *p* < 0.0004. Box plots display 2nd and 3rd quartiles and the median, bars indicate maximum and minimum. See Source Data for raw values and sample numbers. Source data are provided as a Source Data file.

Second-site loss-of-function of the CLE45 receptor BAM3 suppresses the PPSE differentiation failures of *brx* mutants^18,19^, but it remains unclear why this is the case. Partial rescue of *brx* was observed upon *cle45* second-site mutation (Supplementary Fig. 5a, b), corroborating redundancy of CLE45 with other PPSE-specific ‘root-active’ CLE peptides^33^, whose external application also suppresses protophloem differentiation^5,19,21^. However, the disrupted subcellular PIN patterning of *brx*^10^ was largely rescued by either *bam3* or *cle45* second-site mutation (Fig. 3a–e). In contrast to the comprehensive rescue observed in *bam3 brx* double mutants^5,18^, second-site *bam3* mutation surprisingly did not rescue diminished *pax* root growth vigor (Supplementary Fig. 5c) although it rescued visible protophloem differentiation defects (Supplementary Fig. 5d). Moreover, subcellular PIN patterning was not restored in *bam3 pax* double mutants (see below), corroborating our repeated finding that PAX is essential for creating the local PIN minimum in developing PPSEs. In summary, these observations suggest that the impact of CLE45-BAM3 signaling on visible PPSE differentiation defects and subcellular PIN patterning defects can be genetically separated.

Matching the interdependence of BRX and PIP5K polarity, *brx* mutants display increased PI4P levels specifically at the rootward end of developing PPSEs, as indicated by a PI4P-binding fluorescent cellular marker^10,14^. We thus investigated whether CLE45-BAM3 signaling might impact phosphoinositide balance or PAX abundance, and thereby subcellular PIN patterning. Indeed, upon CLE45 treatment, plasma-membrane-association of the PI4P marker increased substantially and specifically in developing PPSEs (Fig. 3f and Supplementary Fig. 5e), in a time frame that had no discernible impact on PPSE identity (Supplementary Fig. 5f). Concomitantly, subcellular PIN patterning was disrupted in a BAM3-dependent manner (Fig. 3g–k) and PAX was displaced from the plasma membrane (Fig. 3l). Similar observations were made with the PPSE-specific root-active CLE26 peptide^19,33,34^, but not with CLE9/10, which is involved in xylem and stomata development^35^, or CLE41/44, which is involved in secondary growth regulation^36,37^ (Fig. 3m and Supplementary Fig. 5g). Consistently, CLE45 treatment also induced both BRX and PIP5K1 plasma-membrane-dissociation (Fig. 3n). Finally, PAX polarity and abundance were largely restored in *bam3 brx* or *brx cle45* mutants (Fig. 3o and Supplementary Fig. 5h). In summary, our results show that short CLE45 treatments recapitulate *brx* protophloem defects in a BAM3-dependent manner and suggest that CLE45-BAM3 signaling interferes with rheostat and PIP5K polarity.

### Effectors of CLE45 signaling directly interact with a rheostat component

**Fig. 4:**
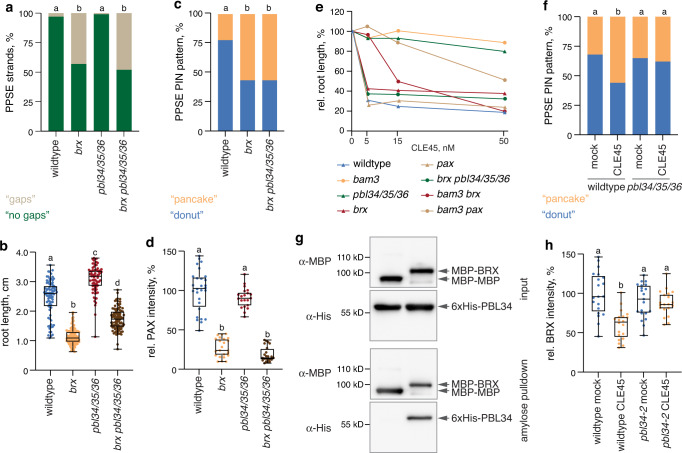
PBL34/35/36 are a branched output of CLE45-BAM3 signaling. **a** Scoring of PPSE differentiation failures (“gaps”) in 7-day-old seedlings of indicated genotypes. *n* = 59–162 PPSE strands; Statistically significant differences (lower case letters) were determined by Fisher’s exact test, *p* < 0.0001. **b** Root length of 8-day-old seedlings of indicated genotypes. The *pbl34/35/36* line is the triple loss-of-function mutant. *n* = 74–121 roots; Statistically significant differences were determined by ordinary one-way ANOVA, *p* < 0.0001. **c** Scoring of subcellular PIN patterning in developing PPSEs of 5-day-old seedlings of indicated genotypes. *n* = 14–26 roots, 149–267 PPSEs per genotype; Statistically significant differences were determined by Fisher’s exact test, *p* < 0.001. **d** Relative abundance of PAX, determined by anti-PAX immunostaining in developing PPSEs of 5-day-old seedlings of indicated genotypes. *n* = 21–28 roots, 182–252 PPSEs per genotype; Statistically significant differences were determined by ordinary one-way ANOVA, *p* < 0.0001. **e** Relative root length of 7-day-old seedlings of indicated genotypes, grown on mock or increasing concentration of CLE45 peptide. *n* = 17–49 roots. **f** Scoring of subcellular PIN patterning in developing PPSEs of 5-day-old wildtype seedlings, 6 h after transfer onto mock or 10 nM CLE45 peptide, determined by anti-PIN1 immunostaining. *n* = 37–38 roots, 421–468 PPSEs per treatment; Statistically significant differences were determined by Fisher’s exact test, *p* < 0.0002. **g** Western blot detection of maltose-binding protein (MBP) fusion proteins and 6x His-tagged PBL34 fusion protein after in vitro pulldown assays with amylose beads (bottom) and its input control (top). **h** Relative abundance of BRX, determined by anti-BRX immunostaining in developing PPSEs of 5-day-old wildtype or *pbl34-2* mutant seedlings, treated with mock or 10 nM CLE45 for 6 h. *pbl34-2* plants express a dominant-negative, kinase-dead PBL34 variant. *n* = 20–21 roots, 205–260 PPSEs per genotype/treatment; Statistically significant differences were determined by ordinary one-way ANOVA, p < 0.0007. Box plots display 2nd and 3rd quartiles and the median, bars indicate maximum and minimum. See Source Data for raw values and sample numbers. Source data are provided as a Source Data file.

Whether CLE45-BAM3 signaling impacts rheostat or PIP5K polarity directly or indirectly remained unclear since no mechanistic link between them is known. Interestingly, triple knock-out of the redundant downstream CLE peptide signaling components PBL34, PBL35 and PBL36 cannot rescue the visible differentiation defects in *brx* mutants (Fig. 4a)^38,39^, but slightly promotes root growth in both wildtype and *brx* background (Fig. 4b). Moreover, neither PIN1 patterning, nor PAX localization was restored in *brx pbl34/35/36* quadruple mutants (Fig. 4c, d). These data suggest that, surprisingly, *brx* is genetically epistatic to *pbl34/35/36*, although *pbl34/35/36* triple mutants are strongly resistant to CLE45^38,39^. This can be understood by our observation that *brx* loss-of-function conferred renewed albeit reduced CLE45 sensitivity both in *bam3 brx* double mutant and *brx pbl34/35/36* quadruple mutant backgrounds (Fig. 4e). Thus alternative, parallel CLE45-sensing pathways appear to be deregulated in *brx*, possibly reflecting redundancy between BAM3 and related receptors in other contexts^21,40,41^. Because higher order mutants of PBL34/35/36-related receptor-like cytoplasmic kinases (RLCKs) do not display any marked resistance to various CLE peptides including CLE45 (Supplementary Fig. 5i)^38^, alternative downstream effectors may also be involved. However, our findings also indicate that PBL34/35/36 signaling represents a branched output of CLE45-BAM3 signaling because *brx pbl34/35/36* mutants were considerably more sensitive to CLE45 treatment than *bam3 brx* mutants (Fig. 4e), and because CLE45-induced disruption of PIN patterning was PBL34/35/36-dependent (Fig. 4f).

Strikingly, we observed specific interaction of BRX with PBL34, PBL35, and PBL36, but not with related RLCKs in yeast-two-hybrid assays (Supplementary Fig. 6a–c). Such interactions were not observed with BRX-LIKE 2 (BRXL2), a partially redundant BRX homolog^10,15^ (Supplementary Fig. 6c). Moreover, no interaction was observed between PBL34/35/36, the BAM3 kinase domain, or the kinase domain of the CLE peptide signaling component CORYNE (CRN)^19^ on the one hand, and PAX, PIP5K, or PDK1 on the other (Supplementary Fig. 6d). BRX and PBL34 co-localize in PPSEs (Supplementary Fig. 7a)^38^, and BRX-PBL34 interaction was further confirmed by in vitro pulldown experiments (Fig. 4g) and bimolecular fluorescence complementation (Supplementary Fig. 7b). Interestingly, BRX does not serve as a good PBL34 substrate in vitro (Supplementary Fig. 7c). However, PBL kinase activity was required for rheostat destabilization, as CLE45-induced BRX plasma-membrane-dissociation was blocked in a dominant-negative kinase-dead *pbl34* mutant (Fig. 4h). Finally, PBL34/35/36 interaction with BRX mapped to the BRX C-terminus (Supplementary Fig. 6e), suggesting that it might compete with PAX and PIP5K. In summary, our data indicate that in the wildtype scenario, CLE45-BAM3 signaling triggers distinct downstream outputs and specifically impairs rheostat polarity through the PBL34/35/36 branch.

### BAM3-mediated signaling antagonizes PIP5K action throughout plant development

**Fig. 5:**
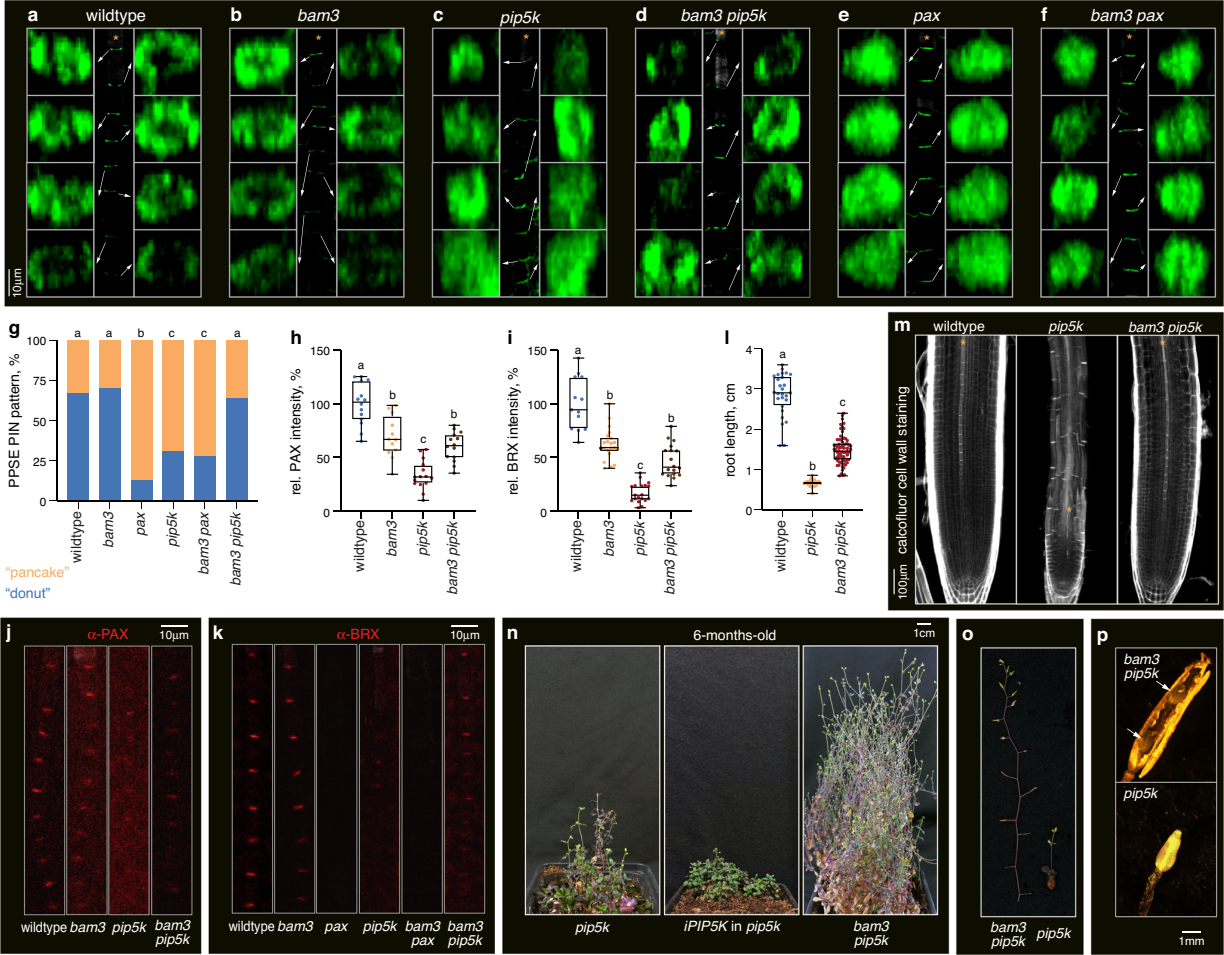
Antagonism between BAM3 signaling and PIP5K determines PAX polarity. **a**–**f** Confocal microscopy images of PIN1 localization (anti-PIN1 immunostaining, green fluorescence, 3D reconstruction) in developing PPSE cell files (calcofluor staining, gray fluorescence; asterisks) of 5-day-old seedlings of indicated genotypes. Center panels: overview; Side panels: magnified images of individual PPSE rootward membranes, with positions indicated by arrows. **g** Scoring of subcellular PIN patterning in developing PPSEs of 5-day-old seedlings of indicated genotypes, determined by anti-PIN1 immunostaining. *n* = 19–51 roots, 210–540 PPSEs per genotype; Statistically significant differences (lower case letters) were determined by Fisher’s exact test, *p* < 0.0001. **h**, Abundance of PAX, determined by anti-PAX immunostaining in developing PPSEs of 5-day-old seedlings of indicated genotypes. *n* = 12–15 roots, 96–135 PPSEs per genotype; Statistically significant differences were determined by ordinary one-way ANOVA, *p* < 0.0005. **i** Abundance of BRX, determined by anti-BRX immunostaining in developing PPSEs of 5-day-old seedlings of indicated genotypes. *n* = 13–21 roots, 130–230 PPSEs per genotype; Statistically significant differences were determined by ordinary one-way ANOVA, *p* < 0.0001. **j** Confocal images of PAX (anti-PAX immunostaining, red fluorescence) in developing PPSEs of 5-day-old seedlings of indicated genotypes. **k** Confocal images of BRX (anti-BRX immunostaining, red fluorescence) in developing PPSEs of 5-day-old seedlings of indicated genotypes. **l** Root length of 7-day-old seedlings of indicated genotypes. *n* = 27–72 roots; Statistically significant differences were determined by ordinary one-way ANOVA, *p* < 0.0001. **m** Confocal images of wildtype, *pip5k* and *bam3 pip5k* mutant root meristems (calcofluor cell wall staining, gray fluorescence). Asterisks mark PPSE cell files. **n** Images of 6-month-old transgenic or mutant plants. **o** Close up images of inflorescence stems from 6-month-old mutant plants. **p** Close up images of siliques from 6-month-old mutant plants. Box plots display 2nd and 3rd quartiles and the median, bars indicate maximum and minimum. See Source Data for raw values and sample numbers. Source data are provided as a Source Data file.

We next asked whether a loss of CLE45-BAM3 signaling could thus compensate for reduced rheostat polarity in *pip5k* mutants. Indeed, second-site *bam3* mutation largely restored PPSE differentiation and subcellular PIN patterning when introduced into the *pip5k* background (Fig. 5a–d, g, Supplementary Fig. 5j). Consistently, this was accompanied by a partial recovery of both BRX and PAX polarity (Fig. 5h–k). In contrast, no significant restoration of PIN patterning or BRX polarity was observed in *bam3 pax* mutants (Fig. 5e–f, k). These results suggest that the self-reinforcing properties conferred to the rheostat by its interaction with PIP5K counteract CLE45-BAM3-PBL34/35/36 signaling and converge on the control of PAX polarity. Moreover, re-emphasizing the central role of protophloem for root development^2,5,25^, *bam3 pip5k* mutants also displayed substantially normalized root meristem organization and growth (Fig. 5l, m). Finally, we observed that unlike iPIP5K1 expression, *bam3* mutation also partially restored the strongly impaired shoot growth of *pip5k* mutants (Fig. 5n), notably stem branching and elongation (Fig. 5o). This might reflect the role of CLE peptide perception by BAM3 and related receptor kinases in different developmental shoot contexts^12,42–44^. For instance, unlike *pip5k* mutants, *bam3 pip5k* mutants also produced few yet viable offspring (Fig. 5p), matching the reported involvement of PIP5K and CLE45 in male gametogenesis and pollen tube growth^45–48^. These observations suggest that BAM3-mediated CLE peptide signaling antagonizes PIP5K action throughout *Arabidopsis* development.

## Discussion

In summary, the contrasting genetic and cell biological scenarios we observed reveal an intricate quantitative interplay that dynamically determines PAX activity and polarity as a key readout required for properly integrated protophloem development^6,9^. *pbl34/35/36* and *bam3* mutants are strongly CLE45-resistant whereas *bam3 brx* double mutants and even more so *brx pbl34/35/36* quadruple mutants are again CLE45-sensitive. We can thus conclude that, besides the CLE45-BAM3-PBL34/35/36 pathway, another CLE45-sensing pathway must be deregulated in *brx* mutant background. This other pathway might employ a BAM3 homolog^41^, or RECEPTOR-LIKE PROTEIN KINASE 2^49^, or STERILITY-REGULATING KINASE MEMBER 1 or 2^48^ as alternative CLE45 receptor(s). Moreover, because multiplexed mutants of other RCLK clades are all CLE45-sensitive, this alternative pathway possibly employs a different class of downstream cytoplasmic effector. From the observation that all *brx* mutant phenotypes are rescued in *bam3 brx* double mutants but not in *brx pbl34/35/36* quadruple mutants, we can conclude that PBL34/35/36 only transduces part of the output of CLE45-stimulated BAM3 activity. The other branch might employ the CLE45 signaling regulator MEMBRANE-ASSOCIATED KINASE REGULATOR 5 (MAKR5), because both root growth and PPSE differentiation failures are largely rescued in *brx makr5* double mutants^50^. Additionally, because CLE45 does not affect PIN patterning or PAX polarity in *bam3* single mutants and *pbl34/35/36* triple mutants, the PBL34/35/36 branch of CLE45-BAM3 signaling apparently interferes with auxin efflux regulation. Moreover, because diminished root growth is not rescued in *bam3 pax* double mutants, we can conclude that PAX-mediated auxin efflux regulation in PPSEs promotes root growth. Yet, the observation that *bam3 pax* double mutants are largely CLE45-resistant (Fig. 4e) and do not display any visual PPSE differentiation failures corroborates that BAM3-mediated CLE45 signaling interferes with PPSE differentiation. In summary, our results not only corroborate that auxin efflux regulation and PPSE differentiation are linked^6,8^, but also reiterate that BRX and PAX polarity are deeply intertwined^10^.

In this context, it is noteworthy that the PPSE differentiation defects in *pax* mutants are quantitatively milder than in *brx* mutants^6^. Given our finding that BRX polarity depends to some degree but not absolutely on PAX polarity, this suggests that the PPSE differentiation failures in *pax* mutants are a consequence of sub-stoichiometric BRX recruitment to the plasma membrane. Likewise, the quantitatively severe PPSE differentiation defects observed in *pip5k* mutants may mainly reflect the dependence of BRX-PAX rheostat polarity on PIP5K. This idea is supported by our finding that the iPIP5K1 variant partially restores rheostat polarity and confers a partial rescue of *pip5k* defects. Moreover, the observation that PIP5K is dispensable for PIN patterning and PPSE differentiation in the absence of BAM3 suggests that in the protophloem context, PIP5K activity is mainly required to promote PAX polarity.

**Fig. 6:**
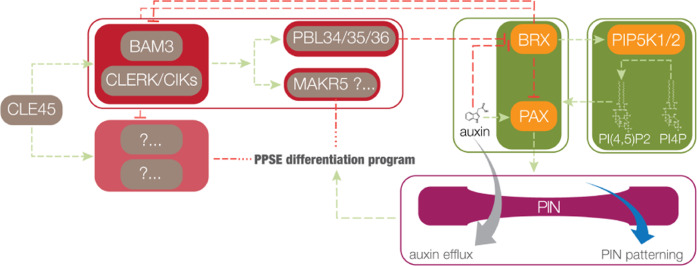
Schematic model of PAX polarity control. Schematic illustration of the central role of PAX polarity and activity for the integration of protophloem sieve element differentiation and root growth (not presenting spatial aspects; boxed in components represent modules rather than cellular compartments). Auxin efflux control by the BRX-PAX rheostat is reinforced through mutual interactions with PIP5K, which is necessary to counteract disruption of rheostat localization by CLE45-BAM3-PBL34/35/36 signaling (which also involves the redundant CLERK/CIK co-receptors). BRX dampens CLE45 signaling through BAM3 as well as an unknown redundant pathway in a yet-to-be-identified manner.

Collectively, our results suggest that, in the wildtype situation, rheostat polarity must be reinforced by PIP5K activity to counterbalance its disruption by BAM3-PBL34/35/36-mediated CLE45 signaling (Fig. 6). Rheostat control of auxin efflux is thus subordinate to antagonism between localized PIP5K activity and a distinct branch of CLE peptide signaling output. Finally, our observation that *bam3* mutation also partially rescues shoot phenotypes of *pip5k* mutants suggests that antagonism between CLE peptide signaling and PIP5K action occurs in various contexts of *Arabidopsis* development.

## Methods

### Plant material and growth conditions

The *Arabidopsis* Columbia-0 (Col-0) accession was the wildtype background for all lines used in this study, the *bam3*, *brx*, *pax*, *pip5k1*, *pip5k2*, *pbl34-2,* and *pbl34/35/36* mutant lines have been described before^6,10,18,38^. The *cle45* mutant was created by the CRISPR/Cas9 technique as described^15^, using guide RNA sequences 5′-TGC CTA AGA GCA GAA ATG TT-3′ and 5′-TAA CTT CCT CGA GAA CGC GT-3′. A mutant that carried a T nucleotide insertion in the start codon site was identified and used in the analyses. For tissue culture phenotyping assays, seeds were surface sterilized and then stratified for 2–4 d in the dark at ca. 4 °C. Seeds were then germinated and grown in continuous white light of ca. 120 μE intensity at ca. 22 °C, on vertically placed Petri dishes that contained 0.5 X Murashige and Skoog (MS) media supplemented with 0.85% agar and 0.3% sucrose. CLE45 peptide (Genscript custom peptide synthesis, ≥ 85% purity), estradiol, brefeldin A (BFA) or 4-amino-1-tert-butyl-3-(1′-naphthyl)pyrazolo[3,4-d]pyrimidine (NAPP) was added where pertinent at indicated concentrations. Adult plants were monitored under controlled conditions in a walk-in chamber (16 h light–8 h dark cycle with ca. 130μE light intensity, ca. 22 °C, ca. 60% humidity).

### Generation of constructs and transgenic lines

Transgenic constructs for plant transformation were created in the pH7m34GW binary vector using the *Gateway*^TM^ cloning technology. All binary constructs were verified by Sanger sequencing and introduced into *Agrobacterium tumefaciens* strain GV3101pMP90 for plant transformation using the floral dip method. For the *PIP5K1::iPIP5K1-CITRINE* and *PIP5K1::PIP5K1-CITRINE* constructs, a 2584 bp promoter fragment upstream of the *PIP5K1* (AT1G21980) start codon of was amplified and cloned into pDONRP4P1R. The genomic *PIP5K1* transcript region was cloned into pDONR221 without its STOP codon. For iPIP5K1, a K536A point mutation was created by site-directed mutagenesis that replaced the AAA codon with a GCA codon. The entry clones were combined with CITRINE in pDONRP2RP3 and then transferred into the destination vector pH7m34GW. The *PAX::ciPAX-CITRINE* construct was cloned with a similar strategy, using the 4500 bp promoter fragment upstream of the *PAX* (AT2G44830) start codon, and site-directed mutagenesis to introduce the M440G point mutation by replacing the ATG codon with a GGG codon. For inducible constructs, the 2,500 bp fragment upstream of the *CVP2* (AT1G05470) start codon was cloned into the p1R4-ML:XVE plasmid^51^ and then combined with genomic fragments of the *PAX*, *BRX* (AT1G31880), *PIP5K1* and *PID* (AT2G34650) transcript regions (amplified without their STOP codons), and fused in frame with CITRINE. Oligonucleotides used for cloning the *CVP2* promoter, for site-directed *PIP5K1* and *PAX* mutagenesis as well as the *iPIP5K1* and *ciPAX* sequences are provided in Supplementary Table 1.

### Protein immunolocalization and confocal imaging

Whole mount immunolocalization in 5-day-old seedlings was performed as previously described^10^. The primary antibody dilutions were: 1:500 for anti-GFP mouse; 1:500 for anti-BRX rabbit; 1:250 for anti-PIN1 goat; 1:2000 for anti-KNOLLE rabbit; 1:250 for anti-PAX rabbit. The secondary antibody dilutions were: 1:500 for Alexa Fluor 488 anti-mouse; 1:500 for Alexa Fluor 546 anti-rabbit; 1:500 for Alexa Fluor 546 anti-goat. Confocal microscopy was performed with Leica SP8, Leica Stellaris, Zeiss LSM 700 and Zeiss LSM 880 with Airyscan inverted confocal scanning microscopes. To visualize reporter genes and staining signals, the following fluorescence excitation-emission settings were used: CITRINE excitation 514 nm, emission 529 nm; Venus/YFP excitation 515 nm, emission 528 nm; propidium iodide (PI) excitation 536 nm, emission 617 nm; Alexa Fluor 488 excitation 498 nm, emission 520 nm; Alexa Fluor 546 excitation 556 nm, emission 573 nm; Calcofluor white excitation 405 nm, emission 425–475 nm. 1 mg/ml 4′,6-diamidino-2-phenylindole (DAPI) was used together with calcofluor white staining to well recognize the different division stages based on the status of nucleus stained by DAPI. Pictures were taken with ×20 or ×40 water/oil immersion objectives. For presentation, composite images had to be assembled in various instances. Sequential scanning was used for colocalization studies to avoid any interference between fluorescence channels. For image and PIN1 patterning analyses, ImageJ, LAS X, Zeiss Zen 2011 (black edition) and Imaris image analysis software were used. For signal quantifications, regions of interest for all samples were analyzed in the same area of the root meristem (i.e., developing protophloem sieve elements before their transition to partial elongation and cellular rearrangements), and the average signal intensity per transgenic line was calculated as the mean of means.

### Bimolecular fluorescence complementation assay

To detect the PBL34-BRX interaction in planta, *35S::PBL34-cYFP* and *35S::nYFP-BRX* were transiently co-expressed in *Nicotiana benthamiana* leaves, and the eYFP signal was detected by confocal microscopy using a Leica SP8 instrument with excitation 506 nm and emission 530 nm. *35S::PBL39-cYFP* was used as a negative control. *35S::BRX-CITRINE*, *35S::PBL34-CITRINE* and *35S::PBL39-CITRINE* were assayed to monitor the localization of the individual proteins.

### Yeast two-hybrid assay

The full-length coding sequences of genes or gene fragments as indicated were cloned and inserted into pGBKT7 or pGADT7 vectors as baits or preys. The Golden Yeast strain was used for yeast two-hybrid assay. Yeast transformation was performed using a transformation kit (Frozen-EZ Yeast Transformation II Kit™, T2001, ZYMO RESEARCH), and interactions were tested on -Trp-Leu and on -Trp-Leu-Ade-His medium. 3-AT was added to selection media for BRX-related bait proteins to suppress auto-activation. Oligonucleotides used for cloning the yeast-two-hybrid constructs are provided in Supplementary Table 1.

### Phenotyping

For root length measurements, plates or seeds were imaged using a high resolution (1’200 dpi) flatbed scanner. Seedling root length was determined with *Fiji* image analysis software (version 2.0.1/1.53i) and suitable plug-ins. For quantification of ‘gap cells’ in protophloem sieve element cell files or visualization of fluorescent protein localization, roots were imaged after calcofluor white or propidium iodide staining by confocal microscopy as described above.

### Recombinant protein expression, purification and in vitro assays

The *PBL34* coding sequence was cloned into the pET28a(+) expression vector at NheI and NotI sites using oligonucleotides 5′-TAG CTA GCA TGG GTT TGG ATG CTG-3′ and 5′-TAG CGG CCG CCT ATG TAG TTG CTC CTT TAG-3′. The *BRX* coding sequence was cloned into the pOPINM vector with InFusion (Takara) using oligonucleotides 5′-AAG TTC TGT TTC AGG GCC CGA TGT TTT CTT GCA TAG C-3′ and 5′-ATG GTC TAG AAA GCT TTA GAG GTA CTG TGT TTG-3′. 6xHis-PBL34 wildtype or kinase-dead (D275A, PBL34*) proteins were expressed in *E. coli* strain BL21(DE3)-VR2-pACYC-LamP. 6xHis-MBP(-MBP) and 6xHis-MBP-BRX proteins were expressed in *E. coli* strain BL21(DE3) Rosetta pLysS. Proteins were purified using HisPur cobalt resin (Thermo). Pulldown and kinase assays were performed as previously described^38^.

### Statistics and reproducibility

Analyses to determine statistical significance were performed in Graphpad Prism software, version 9.3.1. Specific statistical tests used are indicated in the figure legends and were always two-sided. All experiments were replicated at least twice, typically three times.

### Reporting summary

Further information on research design is available in the Nature Portfolio Reporting Summary linked to this article.

## Supplementary information

Supplementary Information

Reporting Summary

## Data Availability

All data in this study is available in the main text or the Supplementary Information. Source data are provided with this paper.

## Source data

Source Data
