# Supplementary material for: A phosphoinositide hub connects CLE peptide signaling and polar auxin efflux regulation
Source: Nat Commun. 2023 Jan 26;14:423. doi: 10.1038/s41467-023-36200-0 (PMC9879999; doi:10.1038/s41467-023-36200-0)
Supplement: Supplementary file 1 — Supplementary Information [file 41467_2023_36200_MOESM1_ESM.pdf]

Supplementary Information

for

A phosphoinositide hub connects CLE peptide signaling and polar auxin efflux regulation

Qian Wang, A. Cecilia Aliaga Fandino, Moritz Graeff, Thomas A. DeFalco, Cyril Zipfel & Christian S. Hardtke

Supplementary Figures 1-7
Supplementary Table 1

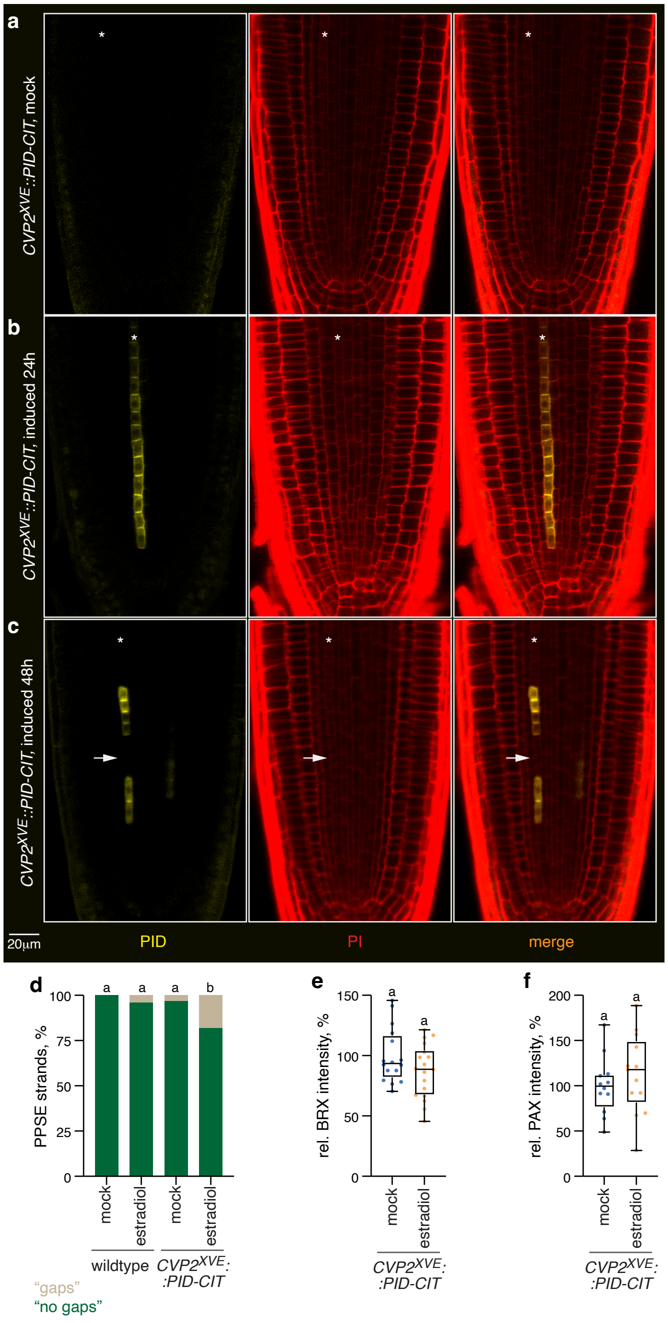

Supplementary Fig. 1 | Perturbation of PPSE development by PIN depolarization. a-c, Confocal live microscopy images of PID-CITRINE fusion protein (left panels, yellow fluorescence) in developing PPSE cell files (asterisks), upon transfer of 5-day-old transgenic seedlings on mock conditions or 5 micromolar estradiol. Arrowheads in (c) point out disappearance of expression indicative of differentiation failure because the *CVP2* promoter is also a marker of PPSE identity. Middle panels: propidium iodide (PI) cell wall staining (red fluorescence); Right panels: overlay; **d,** Scoring of PPSE differentiation failures ("gaps") in 7-day-old wildtype and *CVP2*^{XVE}*::PID-CIT* seedlings 48h after transfer onto mock or 5 micromolar estradiol. n=28-46 PPSE strands; Statistically significant differences (lower case letters) were determined by Fisher's exact test, p≤0.0368. **e-f,** Relative abundance of BRX (e) and PAX (f) as determined by anti-PAX and anti-BRX immunostaining in developing PPSEs of 5-day-old seedlings in mock conditions or after 8h PID-CIT induction on 5 micromolar estradiol. (e): n=16-20 roots, 140-166 PPSEs per treatment; (f): n=10-15 roots, 97-122 PPSEs per treatment; Box plots display 2nd and 3rd quartiles and the median, bars indicate maximum and minimum. See Source Data for raw values and sample numbers. Source data are provided as a Source Data file.

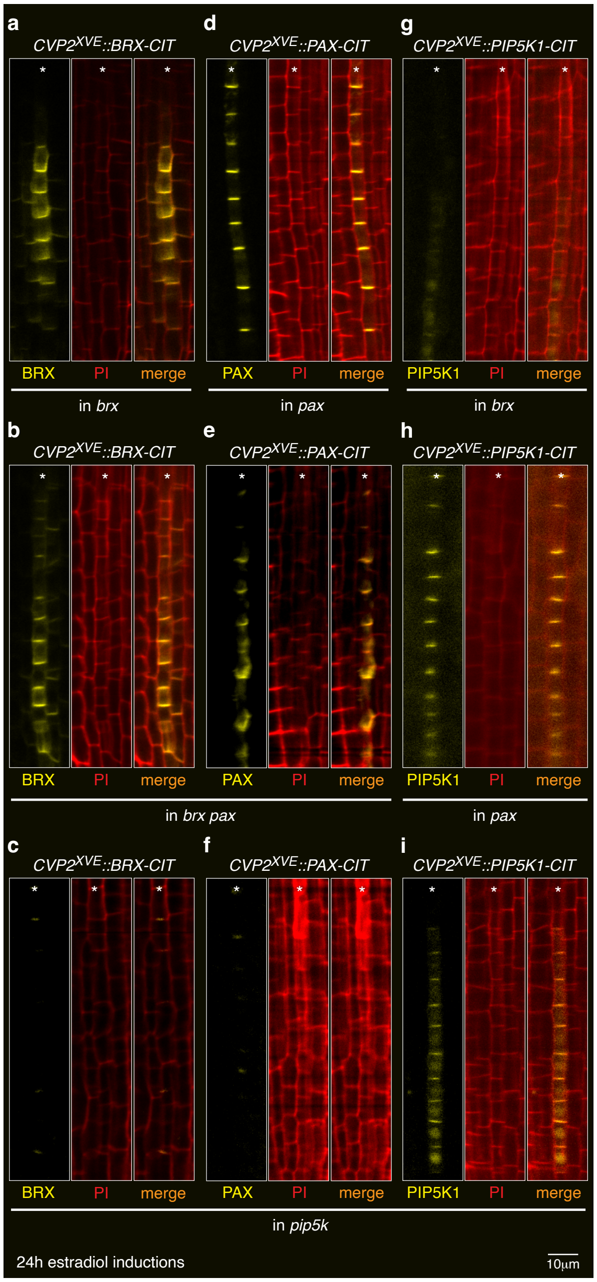

Supplementary Fig. 2 | Interdependence of rheostat and PIP5K polarity in developing protophloem sieve elements. a-i, Confocal live microscopy images of PPSE-specific inducible transgenic BRX, PAX or PIP5K1-CITRINE fusion proteins (left panels, yellow fluorescence) in developing PPSE cell files (asterisks) of indicated mutant backgrounds, upon transfer of respective 6-day-old transgenic seedlings on 5 micromolar estradiol for 24h. Note that compared to endogenous levels, the induced transgenic fusion proteins are over-expressed. Middle panels: propidium iodide (PI) cell wall staining (red fluorescence); Right panels: overlay;

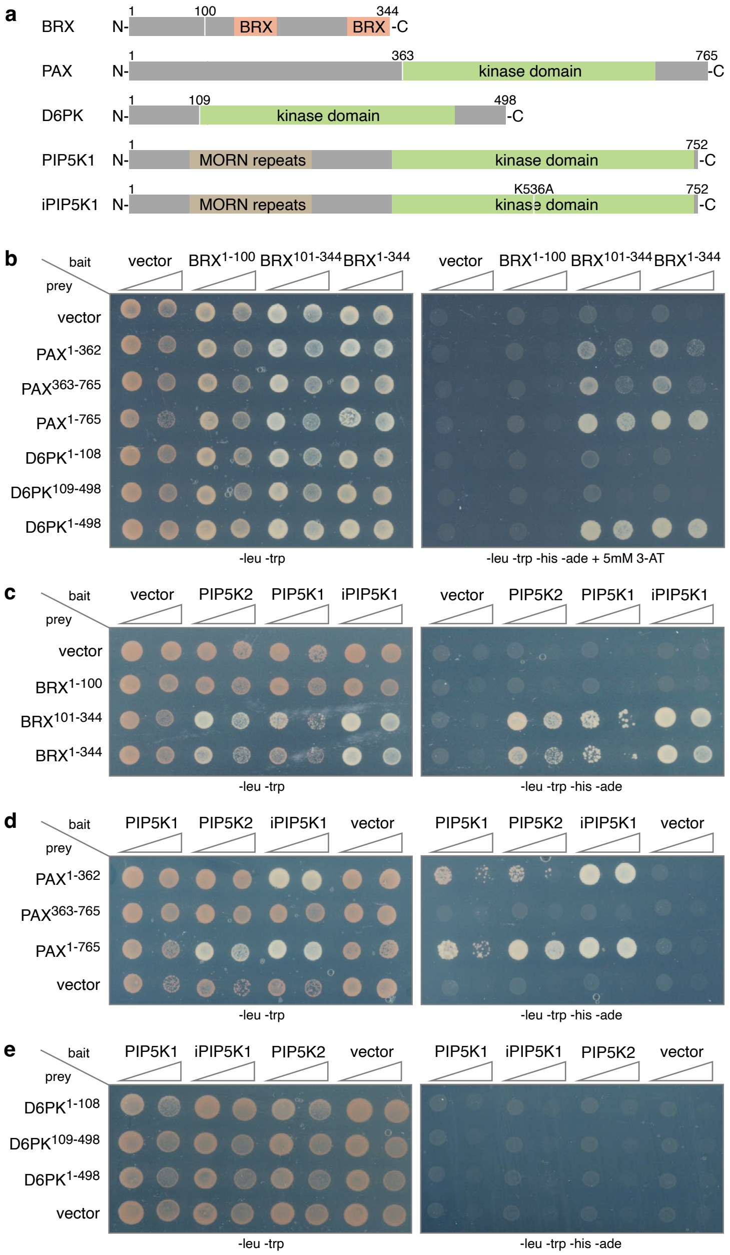

Supplementary Fig. 3 | Protein-protein interactions between the rheostat components and PIP5K. a, Schematic overview of the principal prey and bait proteins scored in yeast-two-hybrid assays. **b-e,** Interaction of indicated bait and prey fusion proteins in yeast-two-hybrid assays, showing comparison of non-selective (left panels) and selective (right panels) media. Note that 3-AT was added to selection media for BRX-related bait proteins to suppress auto-activation (b, e).

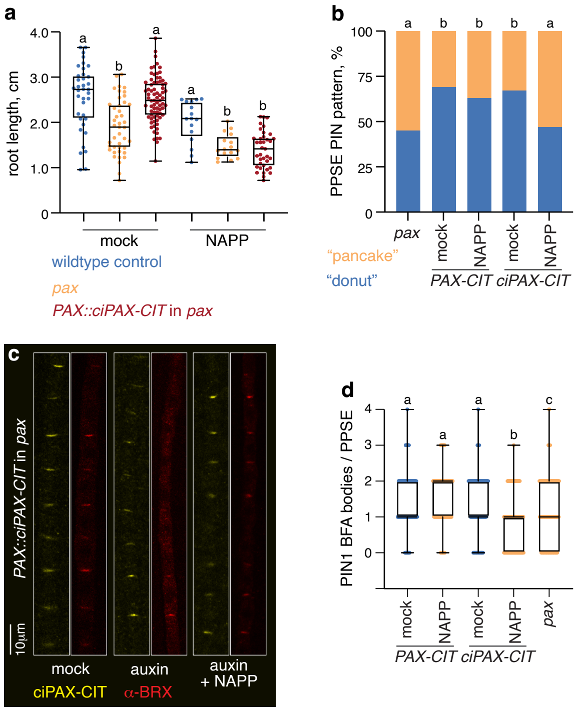

Supplementary Fig. 4 | PAX inactivation perturbs subcellular PIN patterning. a, Root length of 5-day-old wildtype, *pax,* and *PAX::ciPAX-CIT* transgenic seedlings, grown on mock or 2 micromolar NAPP-1 (for ciPAX inhibition). n=16-75 roots; Statistically significant different samples (lower case letters) were determined by ordinary one-way ANOVA, $p<0.003$. **b,** Scoring of subcellular PIN patterning in developing PPSEs of 5-day-old *PAX::PAX-CIT* and *PAX::ciPAX-CIT* transgenics in *pax* mutant background, grown on mock or 2 micromolar NAPP-1 (for ciPAX inhibition), as determined by anti-PIN1 immunostaining. n=19-36 roots, 172-312 PPSEs per genotype/treatment; Statistically significant differences were determined by Fisher's exact test, $p<0.01$. **c,** Confocal microscopy images of BRX (anti-BRX immunostaining, red fluorescence) and ciPAX-CITRINE fusion protein (anti-GFP immunostaining, yellow fluorescence) in developing PPSEs of 5-day-old *PAX::ciPAX-CIT* transgenics in *pax* mutant background, in mock conditions or after 1h auxin (10 micromolar 1-naphthyl-acetic acid) treatment with or without 2 micromolar NAPP-1 (for ciPAX inhibition). **d,** Number of BFA bodies per PPSE in 5-day-old *pax* mutants and *PAX::PAX-CIT* or *PAX::ciPAX-CIT* transgenic seedlings grown on mock or 2 micromolar NAPP-1 (for ciPAX inhibition), and then transferred onto 50 micromolar BFA (mock). n=15-19 roots, 167-272 PPSEs per genotype; Statistically significant different samples were determined by ordinary one-way ANOVA, $p\leq0.0012$. Box plots display 2nd and 3rd quartiles and the median, bars indicate maximum and minimum. See Source Data for raw values and sample numbers. Source data are provided as a Source Data file.

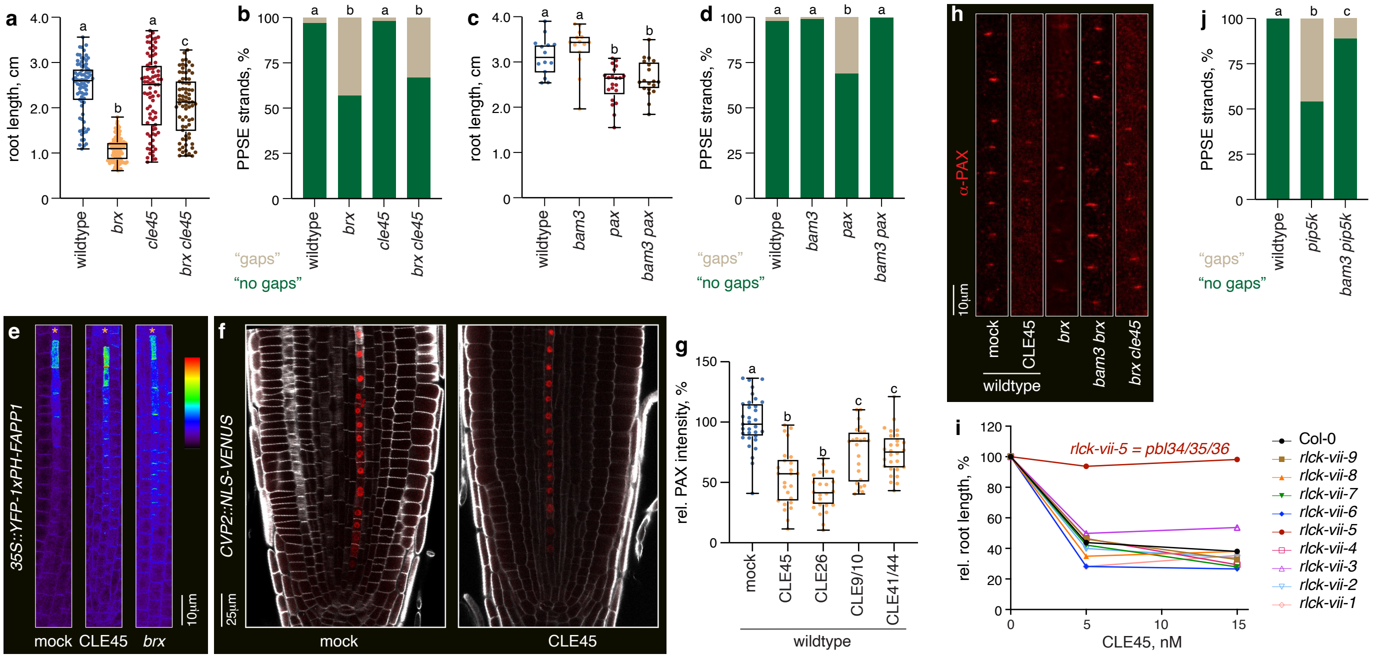

Supplementary Fig. 5 | Loss of CLE45-BAM3 signaling suppresses *brx*, but not *pax* mutant phenotypes. a, Root length of 7-day-old wildtype, *brx*, *cle45* and *brx cle45* seedlings. n=73-119 roots; Statistically significant different samples (lower case letters) were determined by ordinary one-way ANOVA, p≤0.0371. **b,** Scoring of PPSE differentiation failures ("gaps") in 7-day-old wildtype, *brx*, *cle45* and *brx cle45* seedlings. n=59-162 PPSE strands; Statistically significant differences were determined by Fisher's exact test, p<0.0001. **c,** Root length of 7-day-old seedlings of wildtype, *bam3*, *pax*, and *bam3 pax* mutant seedlings. n=14-21 roots; Statistically significant differences were determined by ordinary one-way ANOVA, p≤0.0022. **d,** Scoring of PPSE differentiation failures ("gaps") in 7-day-old wildtype, *bam3*, *pax*, and *bam3 pax* mutant seedlings. n=42-131 PPSE strands; Statistically significant differences were determined by Fisher's exact test, p=0.0033. **e,** Confocal microscopy images of the constitutively expressed PI4P marker YFP-1xPH-FAPP intensity (color scale, purple: lowest; red: highest) in developing PPSEs of 5-day-old seedlings in wildtype treated with mock or 10nM CLE45 for 2h, or *brx*, 5 representative PPSE strands (asterisks) each. **f,** Confocal microscopy images of the PPSE-specific *CVP2::NLS-VENUS* marker (red fluorescence) in wildtype background, in mock conditions or after 6h treatment with 10nM CLE45. Grey fluorescence; calcofluor cell wall staining. **g,** Relative abundance of PAX, determined by anti-PAX immunostaining in developing PPSEs of 5-day-old wildtype seedlings treated with mock or 10nM of indicated CLE peptide for 6h. n=24-37 roots, 241-326 PPSEs per genotype or treatment; Statistically significant differences were determined by ordinary one-way ANOVA, p<0.0004. **h,** Confocal microscopy images of PAX (anti-PAX immunostaining, red fluorescence) in developing PPSEs of 5-day-old seedlings of indicated genotypes, wildtype treated with mock or 15nM CLE45 for 21h. **i,** Relative root length of 8-day-old seedlings of indicated genotypes (multiple mutants for each distinct RLCK clade), grown on mock or increasing concentration of CLE45 peptide. n=13-23 roots. **j,** Scoring of PPSE differentiation failures ("gaps") in 7-day-old wildtype, *pip5k*, and *bam3 pip5k* mutant seedlings. n=47-110 PPSE strands; Statistically significant differences were determined by Fisher's exact test, p≤0.0144. Box plots display 2nd and 3rd quartiles and the median, bars indicate maximum and minimum. See Source Data for raw values and sample numbers. Source data are provided as a Source Data file.

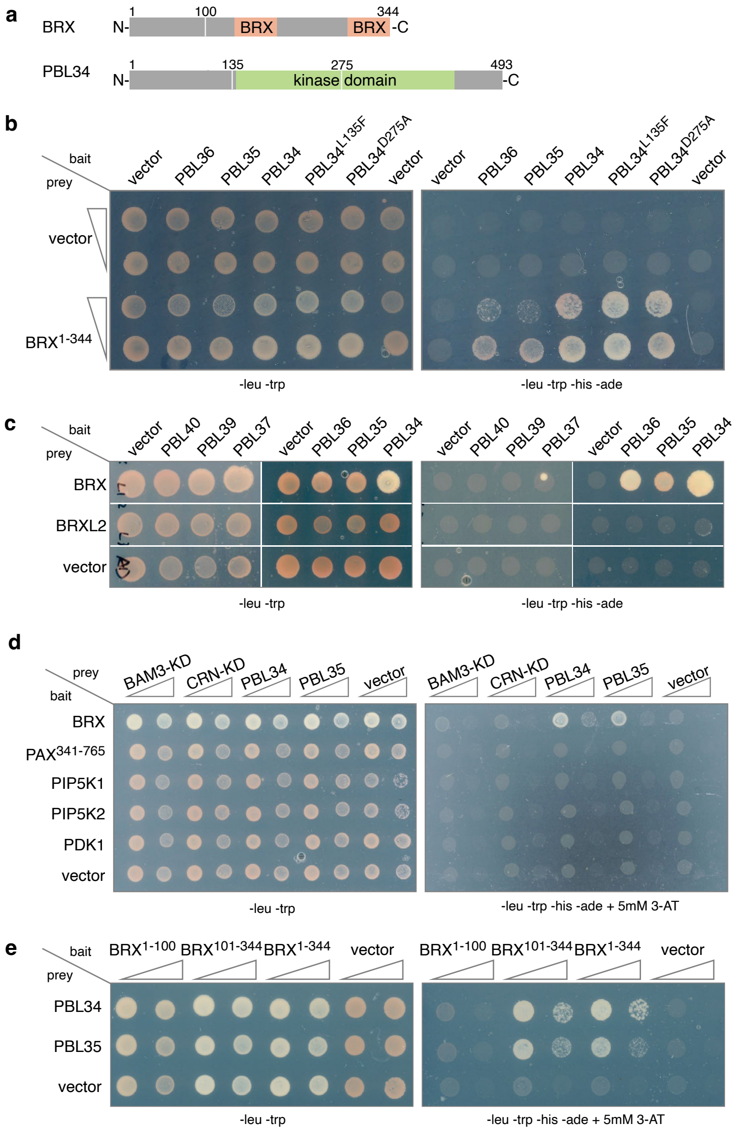

Supplementary Fig. 6 | Yeast-two-hybrid protein-protein interactions between PBL34/35/36 and BRX. a, Schematic overview of the principal prey and bait proteins scored in yeast-two-hybrid assays. **b-e,** Interaction of indicated bait and prey fusion proteins in yeast-two-hybrid assays, showing comparison of non-selective (left panels) and selective (right panels) media. Note that 3-AT was added to selection media for BRX-related bait proteins to suppress auto-activation (d, e). PBL34^{L135F} and PBL34^{D275A} are kinase dead PBL34 versions.

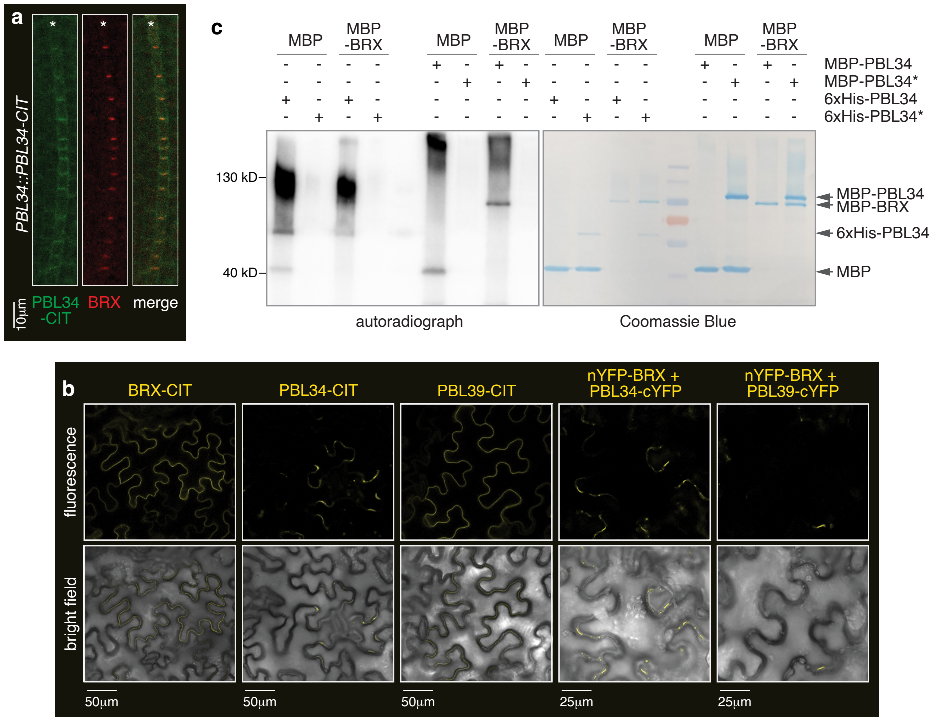

Supplementary Fig. 7 I Protein-protein interactions between PBL34 and BRX. a, Confocal microscopy images of *PBL34::PBL34-CITRINE* transgenics, after simultaneous immunodetection of the fusion protein (anti-GFP, green fluorescence, left panel) and endogenous BRX (anti-BRX, red fluorescence, middle panel), showing co-localization (right panel) in developing PPSEs of 5-day-old seedlings. Asterisks mark the PPSE cell file. **b,** Bimolecular fluorescence complementation assayed in transiently transformed *Nicotiana benthamiana* leaves. Note the more patchy plasma membrane association of PBL34 fusion protein as compared to BRX or the negative control, PBL39. **c,** In vitro kinase assays with MBP-tagged or 6x His-tagged PBL34 kinase, and MBP or MBP-BRX fusion protein as substrate. A kinase-dead inactive PBL34 version (PBL34*) was used as negative control.

Supplementary Table 1

DNA sequences

Oligonucleotide sequences (5`-3`)

Oligonucleotides used for cloning the estradiol-inducible CVP2 promoter:

pXVE_pCVP2_1F A	CGATACCGGTGGTACCCAATCTTATTTATCCTTGACTGAAAGTG
pCVP2_XVE_1R	GCCCGGAATTGGTACGTACTGTTGCTTCTTCTCTGCAAGTG

Oligonucleotides used for site-directed mutagenesis:

PIP5K1_K536A_F	GACCTCGCAGGGTCTTCTCATGGGCG
PIP5K1_K536A_R	AGACCCTGCGAGGTCAAACCGTCTCTG
AGC1-3_M440G_fw	GGGGAATACTGTCCTGGAGGT
AGC1-3_M440G_rew	GACCAAACACGAGAATCTGTCAG

Oligonucleotides used for cloning coding regions in the yeast-two-hybrid system:

Y2H-BRX-F	CTGCATATGATGTTTTCTTGCATAGCT
Y2H-BRX-R	CGGGAATTCTTAGAGGTACTGTGTTTG
Y2H-BRX 100aa-R	CGGGAATTCTTAGTTTGTGAAGTCCCAAGC
Y2H-BRX 101aa-F	CTGCATATGTCCTCTCATCATCCAGCT
Y2H-PAX-F	CTGCATATGATGCTGGAAATGGAAAGA
Y2H-PAX-R	CGGGAATTCTTAGAAAAACTCAAAGTC
Y2H-PAX 362aa-R	CGGGAATTCTTAGTGGCTCATTCCCAAAATCCC
Y2H-PAX 363aa-F	CTGCATATGTTCAAGTTGCTGAAACGA
Y2H PIP5K1-F	CTGCATATGATGAGTGATTCAGAAGAA
Y2H PIP5K1-R	GATCCCCGGGTTAGCCCTCTTCAATGAA
Y2H PIP5K2-F	CTGCATATGATGATGCGTGAACCGCTT
Y2H PIP5K2-R	GATCCCCGGGTTAGCCGTCTTCGATGAA
Y2H-D6PK-F	CTGCATATGATGATGGCTTCAAAAACTCCA
Y2H-D6PK 108aa-R	CGGGAATTCTCAATGATTCAAACCCAAACCACC
Y2H-D6PK 109aa-F	CTGCATATGTTTAGGCTCTTGAAGAGG
Y2H-D6PK-BD-R	CCGCTGCAGTCAGAAGAAATCAAACTCAAG
Y2H-D6PK-AD-R	CAGCTCGAGTCAGAAGAAATCAAACTCAAG
Y2H-BAM3 KD-F	CTG CAT ATG GTC AAG AAT AGG AGA ATG AGA
Y2H-BAM3 KD-R	CGG GAA TTC TTA GAA AGT ATT AGG CTG TTT
Y2H-PBL34-F	GCCGAATTCATGGGTTTGGATGCTGTT
Y2H-PBL34-R	GACGGATCCCTATGTAGTTGCTCCTTT
Y2H-PBL35-F	CTGCATATGATGGGTTTCGATTCTGTT
Y2H-PBL35-R	CGGGAATTCCTAAGTAGTTGCACCTTT
Y2H-PBL36-F	CTGCATATGATGGCTACAAAGTTAGAG
Y2H-PBL36-R	CGGGAATTCTCATGGCTCTTTTCCTTT
Y2H-CRN KD-F	CTGCATATGTTGGTTCGTAGCATTGTC
Y2H-CRN KD-R	CGGGAATTCCTAAAAGCTGTGCAGTTG
Y2H-PDK1-F	GCCGAATTCATGTTGGCAATGGAGAAA
Y2H-PDK1-R	GACGGATCCTCAGCGGTTCTGAAGAGT
Y2H PBL37-F	GCCGAATTCATGAAGTGTTTTCACTTCAC
Y2H PBL37-R	GACGGATCCCTACCATGTTCTGACCAGTC
Y2H PBL39-F	GGGGATCCGTATGAAGTGTTTCTTGTTCTC
Y2H PBL39-R	CCGCTGCAGTCAACAAGCTCTTATTGTCT
Y2H PBL40-F	CTGCATATGATGAAATGCTTCTTATTCCC
Y2H PBL40-R	CGGGAATTCTCAACAAGCTCTCACATTCT
Y2H PIP5K5 F	CTGCATATGATGAGCAAGGACCAAAGCTA
Y2H PIP5K5 R	GATCCCCGGGTCAATTGTCATCTGTGAAGA
Y2H PIP5K7 F	ATGGCCATGGAGGCCATGGATATGAGGTCTGGAG
Y2H PIP5K7 R	GACGGATCCCTACCTTTCTTCTGGGAACAC

DNA sequences (5`-3`)

iPIP5K (K536A mutant variant):

ATGAGTGATTCAGAAGAAGACGAAGAAGAAGAAGAAGCTAGTGAAGTCATTTTAAGCAGCGTCGTTCAGAAGAAGAAGAAGAAGAATCTGCGTTTC
GGTGAAGAAGTAGAGAGACGAGACGGTTTGGTGTTGTTAGCTCAATCTACTCCAATGGTGAGATCGAGATCTCAAGGAACGACTCGGCGCGTGACT
CCCACGCCTTTAGTAGATGTTGAGAAGCCGCTACCAAACGGAGATCTTTACATCGGAAGTTTCTCCGGTGGGTTTCCACATGGATCCGGGAAGTATC
TATGGAAAGATGGGTGCATGTACGAAGGAGATTGGAAACGAGGGAAAGCTTCAGGGAAAGGCAAATTCTCATGGCCAAGTGGAGCTACTTACGAA
GGTGAATTCAAATCTGGGAGAATGGAAGGTTTTGGTACTTTCACTGGAGCTGATGGAGATACTTATAGAGGAACTTGGGTTGCTGATAGAAAACAC
GGACATGGACAAAAGCGTTATGCAAATGGAGATTTTTATGAAGGTACTTGGAGAAGGAATCTACAAGATGGTAGAGGTAGGTATGTGTGGAGAAAT
GGGAATCAGTATACTGGAGAGTGGCGTAGTGGTGTGATTTCTGGGAAAGGTTTGCTTGTTTGGCCTAATGGGAATCGATATGAAGGTTTGTGGGAG
AATGGGATTCCTAAAGGGAATGGTGTGTTTACTTGGAGTGATGGGAGTTCTTGTGTTGGTGCTTGGAATGAGAGTAATATTATGAGGAGTTTCTTTA
ATGGTGTTGAGAAGAATGATTTGATTGTGGGGAATAGGAAGAGATCTTCTGTTGATAGTGGAGCTGGGAGTTTGGGAGGTGAGAAAGTTTTTCCAA
GAATCTGTATTTGGGAATCTGATGGTGAAGCTGGAGATATCACTTGTGATATTATTGATAATGTTGAAGCTTCCATGATTTATAGAGATAGGATCTCT
GTTGATCGTGATGGGTTTAGGCAGTTTAAGAAGAATCCTTGTTGGTTTAATGGTGAGGCTAAGAAACCTGGACAGACTATTTCTAAAGGGCATAAGA
AATATGATTTGATGCTGAATTTGCAATTAGGAATCAGGTATTCTGTTGGCAAACATGCTTCGATTGTTCGTGATCTTAAACAGACTGATTTCGATCCAA
AGGAGAAATTTTGGACAAGGTTTCCACCAGAAGGGACTAAGACAACACCGCCGCATCAGTCAGTGGATTTCAGGTGGAAGGATTATTGCCCTTTAGT
GTTCAGACGTCTCAGGGAGCTTTTCCAAGTGGATCCAGCTAAGTATATGCTAGCTATATGTGGAAATGATGCTCTCCGCGAACTATCTTCACCTGGAA
AGAGTGGAAGCTTTTTTTACCTAACTCAAGATGATAGATTTATGATCAAAACGGTGAAGAAATCAGAAGTCAAGGTTCTTCTAAGAATGCTTCCAAGT
TACTACAAACATGTCTGCCAATACGAAAACTCCCTTGTGACTAGATTCTACGGTGTTCATTGTGTCAAACCTGTTGGTGGCCAAAAGACTCGGTTTATC
GTTATGGGAAACTTATTCTGCTCCGAGTATAGAATCCAGAGACGGTTTGACCTCGCAGGGTCTTCTCATGGGCGTAGCACTGCAAAGCCTGAAGGAG
AAATTGATGAGACCACGACTCTCAAGGACCTTGATCTCAATTTTTCTTTCCGTCTTCAGAGAAACTGGTATCAAGAGCTTATGAAGCAAATAAAACGC
GATTGTGAGTTCTTGGAAGCAGAGAGAATAATGGATTATAGCCTTTTAGTTGGTGTTCACTTCCGTGATGACAACACAGGAGAAAAGATGGGGCTTT
CTCCATTCGTTTTGAGATCTGGTAGGATAGATTCATATCAGAATGAAAAATTCATGCGCGGTTGTCGCTTCCTAGAGGCAGAACTTCAAGACATGGAC
CGGATTTTAGCTGGCAGGAAACCATCGATCAGATTAGGCGCAAACATGCCAGCAAAAGCTGAACGAATGGCCCGGAGAAGCGATTTTGATCAGTAT
TCATCAGGAGGAGCCAGTTATCCATCACACGGTGAGATGTACGAAGTTGTTCTCTACTTTGGAGTCATTGACATCTTGCAAGACTACGACATAACCAA
AAAGATCGAGCATGCGTATAAGTCACTGCAAGCCGATCCTGCTTCGATCTCAGCTGTTGATCCTAAACTCTATTCAAAGAGGTTCAGAGACTTCATCA
GTAGGATCTTCATTGAAGAGGGCTAA

iPAX (M440G mutant variant):

ATGCTGGAAATGGAAAGAGTTGCTGAGCTCAAGAGACTTCCTAGTAAAGGTCCTGTCTCTGGTCACTTATCAAGAAGACCATACTTAGACTTTGAAA
CTAGAGATGCCCCGGGTATGCATTTGGAGAGTTTGAGGGAACGAGCTGCTCGATACAACACGGGAAGATCTGTGAATCCAACTACGACATTGGGGA
GAGAACTAAGCCAAGTTCTGAATGTGCATAGGGAAGATATGATGATGACTCAGTTTGGTGGTAACATGAATGATTTTCAGGAGTTTGAGCCTGTTGT
ATCGTCTGTTAGAACGATGAAGGCCAAGTATCCTTTGTTGGAGATTGAAGAAATTGGAGCTGCTGATGATGATGTTACTTGTAAGGGAAGCAATGAT
ATGTCTGAGGAAGCTGGTTCTAGCTCCTTCCGTGGAGTTAGTCATCCTCCAGAGCCTGACGATATGGATCTAATAACAACTGTTTATGTGCCCATCAG
CGAGAAAAACAAACCTGATTCGGTTTGCTTGATGAAGAGCATGTCTACTACTAAAGGACCCTTTATCGAGGATATTTCGCTCTGTGTGCCTCCAAAGA
AGCCAAGCCCGAGAGTACTTTCACCTGCAGAAAGCATAGTTGAGGAACCTGCTACATCGCTGTCCCCGTTCTCTGTGGCTCGTGCATCGCAGAACAC
TGAAAACTCTCTGCTACCACCAGATTCAGACAAAGAATGTGTTTGGGATGCTTCTCTGCCTCCCAGTACCAATGTGAGCCCACATAGCAGTAGTGTTG
AAAGTATGAATTTGGCTCGGGCTATGAGTATTGCTAATAGCTCTTCTGCAACAAGTACTACTCAGCGGAGCGATGTTGTGCTTAGTATGGACAAGAA
CTACTTTGACAGGAGTATCAGTATGGTTTTGGATTCGTTTGAAAGCACCAAGACCAGTGCAAGCAGAGCAAGTGATAGTAGCGGCCTAAGCGAAGA
GAGCAGCTGGAGCAATTTCACGGGAAGCCTTAATAAGCCACACAAAGGGAATGATCCTTGGTGGAATGCTATCTTGGCTATCCGAACCCGAGATGG
GATTTTGGGAATGAGCCACTTCAAGTTGCTGAAACGATTAGGTTGTGGTGATATTGGGAGTGTCTATCTGGCTGAATTAAGCGGAACTCGATGCCAT
TTTGCTGTGAAAGTCATGGATAAAGCGTCTCTTGAGGACCGGAAGAAGTTGAATCGAGCTCAGACCGAGAGGGATATTCTACAACTATTGGATCATC
CGTTTCTACCGACATTGTACACTCATTTTGAGACTGACAGATTCTCGTGTTTGGTCGGGGAATACTGTCCTGGAGGTGATCTGCACACTCTAAGGCAA
CGTCAACCCGGGAAGCATTTCTCGGAGTACGCTGCTCGATTTTACGCTGCAGAGGTGTTGCTAGCACTAGAGTATCTCCACATGCTCGGTGTTGTTTA
CAGAGACTTGAAGCCTGAGAATGTTCTGGTTCGAGATGATGGTCACATAATGCTTTCAGACTTTGATCTCTCCTTGAGGTGCGCGGTTTCGCCAACAC
TGATCAAAACATTCGACTCCGATCCATCTAGACGAGGCGCATTCTGCGTTCAACCTGCTTGTATGGAGCCTACATCAGCTTGCATCATTCAACCCTCAT
GCTTCTTACCGCGCAGCATCTTCCCTAACAAAAACAAAAAAAACAAGTCCCGTAAAACCCAGGCGGATTTCTTCAAATCACACTCTGGTTCTCTCCCAG
AGCTAGTAGCTGAACCTAACACACGGTCCATGTCCTTTGTTGGAACCCACGAGTACTTAGCTCCAGAGATCATCAAAGGAGAAGGACATGGAAGCGC
AGTGGATTGGTGGACTTTTGGTATCTTTGTGCATGAGCTCCTATATGGGAAAACCCCGTTTAAAGGATCAGGAAATCGAGCTACTCTGTTCAATGTAG
TCGGCGAACAGTTGAAATTCCCCGAGTCACCAGCAACTAGCTATGCAGGCAGGGACTTGATACAGGCTTTACTGGTGAAAGATCCAAAGAACAGGT
TAGGGACAAAGAGAGGAGCAACGGAGATAAAGCAGCATCCATTCTTTGAAGGTGTGAATTGGGCATTGATAAGGTGTAGCACTCCACCTGAAGTAC
CGAGACAGATGGAGACCGAACCGCCACCAAAGTATGGACCGATTGATCCGGTTGGGTTTGGTAGTAATAGCAAAAGGATGATGGGACCACCAGCA
GTATCAGCAGCAGCAGCAGACACGAAATCTGGTGGTAAATTTCTAGACTTTGAGTTTTTCTAA
